# Cracking the chaperone code through the computational microscope

**DOI:** 10.1016/j.cstres.2024.08.001

**Published:** 2024-08-12

**Authors:** Federica Guarra, Cristiano Sciva, Giorgio Bonollo, Chiranjeevi Pasala, Gabriela Chiosis, Elisabetta Moroni, Giorgio Colombo

**Affiliations:** 1 Department of Chemistry, University of Pavia, Pavia, Italy; 2 Department of Medicine, Memorial Sloan Kettering Cancer Center, New York, NY 10065, USA; 3 Chemical Biology Program, Memorial Sloan Kettering Cancer Center, New York, NY 10065, USA; 4 Institute of Chemical Sciences and Technologies (SCITEC) - Italian National Research Council (CNR), Milano, Italy

**Keywords:** Dynamics, Chaperone code, Hsp, Drug development, Functional assemblies

## Abstract

The heat shock protein 90 kDa (Hsp90) chaperone machinery plays a crucial role in maintaining cellular homeostasis. Beyond its traditional role in protein folding, Hsp90 is integral to key pathways influencing cellular function in health and disease. Hsp90 operates through the modular assembly of large multiprotein complexes, with their composition, stability, and localization adapting to the cell's needs. Its functional dynamics are finely tuned by ligand binding and post-translational modifications (PTMs). Here, we discuss how to disentangle the intricacies of the complex code that governs the crosstalk between dynamics, binding, PTMs, and the functions of the Hsp90 machinery using computer-based approaches. Specifically, we outline the contributions of computational and theoretical methods to the understanding of Hsp90 functions, ranging from providing atomic-level insights into its dynamics to clarifying the mechanisms of interactions with protein clients, cochaperones, and ligands. The knowledge generated in this framework can be actionable for the design and development of chemical tools and drugs targeting Hsp90 in specific disease-associated cellular contexts. Finally, we provide our perspective on how computation can be integrated into the study of the fine-tuning of functions in the highly complex Hsp90 landscape, complementing experimental methods for a comprehensive understanding of this important chaperone system.

## Introduction

Cells need to rely on the correct folding of a myriad of proteins to survive in their normal environments and when exposed to internal and external stressors.^1^ The native and functional structure of proteins in the crowded environment of the cell interior is safeguarded by a class of proteins named molecular chaperones,^2^ which oversee the folding, refolding, stabilization, activation, and transport of a large number of substrates, named “clients.”

Chaperones have been investigated over the last 40 years largely by experimental structural, biochemical, and cell biology approaches.^2^, ^3^, ^4^, ^5^, ^6^, ^7^, ^8^, ^9^, ^10^, ^11^ Studies have focused mainly on chaperone activities, the role of enzymatic ATPase in functional regulation, and analysis of *in vitro* folding mechanisms. Lately, the cryo-EM revolution has started to unveil the structural details of the multiprotein complexes that need to be assembled to guide clients to their folded states.^12^, ^13^, ^14^, ^15^, ^16^ In parallel, the explosion of proteomic technologies revealed that a large number of post-translational modifications (PTMs) on chaperones (e.g., phosphorylation, acetylation, methylation, SUMOylation, ubiquitination, etc.)^17^, ^18^, ^19^, ^20^ modify and fine-tune chaperone activities and interactions in health and disease. The emerging view is that chaperones are a class of proteins that, rather than being structurally and functionally homogeneous, display differential activities that depend on the specific environment and conditions. The structures and functions of chaperones transcend the classical view of dynamic assemblies that fold proteins or mediate the assembly of protein complexes. This evolving knowledge encourages us to embrace the complexity of chaperones, which not only uncovers new biology but also provides avenues for therapeutic development by targeting their specific forms and activities, rather than viewing chaperones as uniform entities.

**Fig. 1:**
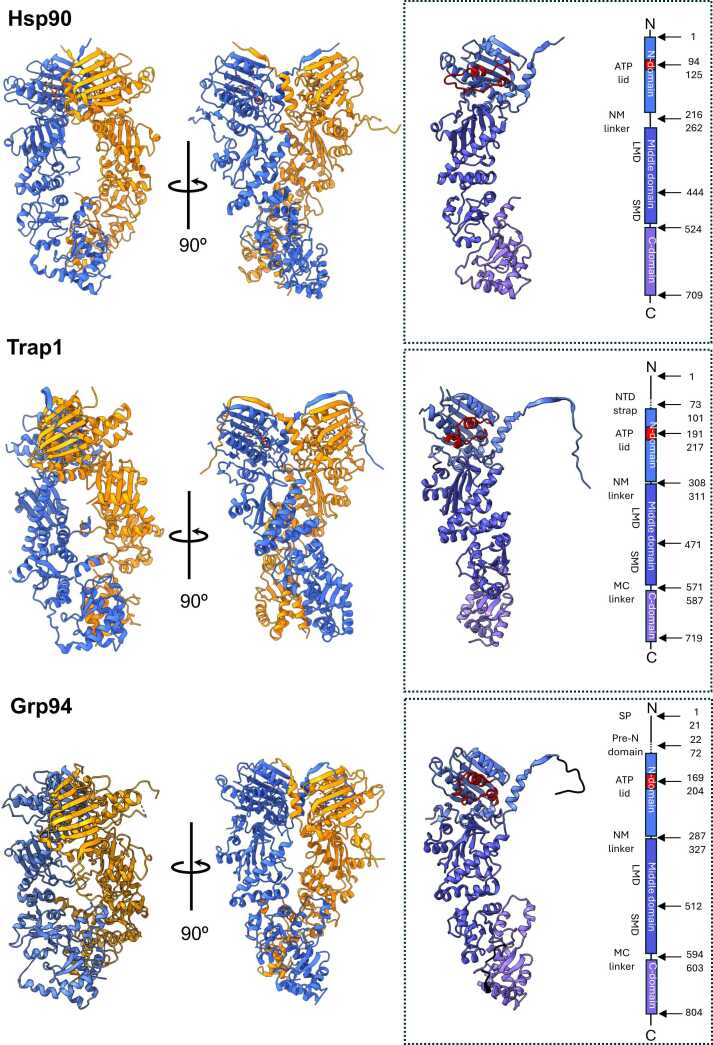
The three-dimensional structure of Hsp90 family proteins. The complete 3D structure obtained from X-ray crystallography for (from top to bottom) cytosolic Hsp90, mitochondrial Trap1, and the endoplasmic reticulum paralog Grp94. The domain and sequence organization are also reported. Structures are reproduced from PDB codes 2cg9 (Hsp90), 4IPE (Trap1), and 5ULS (Grp94). Abbreviation used: Hsp90, heat shock protein 90: PDB, Protein Data Bank.

Heat Shock Protein 90 kDa (Hsp90) is a paradigmatic example of what is reported above. Hsp90 is, in fact, an essential molecular machinery for cell development and maintenance that works late in the folding process to activate a broad array of clients, many of which are involved in oncogenesis. Hsp90 exists in different isoforms: Hsp90 α and β in the cytosol, Grp94 in the endoplasmic reticulum (ER), and Trap1 in mitochondria (Figure 1). From a structural point of view, all isoforms are homodimers with each individual chain consisting of three globular domains: the N-terminal domain (NTD), middle domain, and C-terminal domain (CTD) (Figure 1). X-ray, small-angle X-ray scattering, and kinetic measurements have led to a mechanistic model in which ATP binding at the NTD^21^ shifts the chaperone to a partially closed and then into an asymmetric closed conformation that is significantly strained at the middle domain:CTD interface, the region involved in client binding.^22^, ^23^, ^24^, ^25^, ^26^ Upon ATP hydrolysis, strain is relieved through rearrangement of the client binding-site residues, driving structural changes in the client. In this framework, ATP hydrolysis is coupled to client remodeling.

**Fig. 2:**
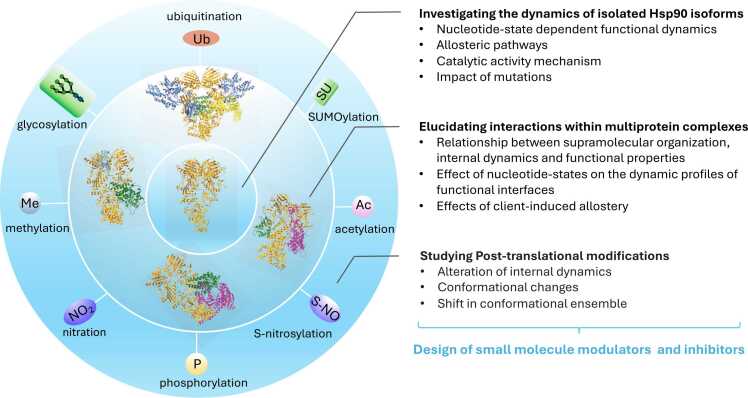
Schematic representation of Hsp90 levels of functional regulation that can be investigated through the computational microscope. The scheme depicts the different layers of functions, interactions, their modulations by ligands, protein–protein interactions, and post-translational modifications, and the insights that can be obtained thanks to computational methods. Abbreviation used: Hsp90, heat shock protein 90.

The functions of Hsp90 are finely modulated by protein–protein interactions with clients and cochaperones (Figure 2). The majority of identified clients of cytosolic and extracellular Hsp90 are related to signal transduction, cell maintenance, growth, and invasiveness and include steroid hormone receptors, kinases, and matrix metalloproteinase proteins.^27^ In the case of ER, Grp94 clients include immunoglobulin Gs and Toll-like receptors.^28^ In the case of the mitochondrial Hsp90 homolog, Trap1, the validated list of clients is rather small and mostly related to mitochondrial protein homeostasis and respiration.^7^, ^29^ The recruitment of chaperones and cochaperones, including Heat Shock Protein 70 kDa (Hsp70), Cdc37, Aha1, Hop, and p23,^27^ provides an additional layer of regulation by structurally organizing complexes for client activation (e.g., Cdc37 for kinases) and by enhancing (e.g., Aha1) or slowing (e.g., p23) ATPase rates: Mechanistically, cochaperones select stochastically distributed Hsp90 conformers that meet functional needs. Aha1 and Cdc37 are overexpressed in cancer and can be post-translationally modified by Hsp90 client enzymes, generating reciprocal regulatory mechanisms of the chaperone machinery: In this context, pharmacological inhibition of clients or downregulation of cochaperone levels hypersensitize cancer cells to Hsp90 inhibitors.

Recent findings indicate that Hsp90 acts as a nucleating site for the assembly of the epichaperomes,^30^, ^31^, ^32^, ^33^ networks of stable, survival-facilitating multiprotein complexes characterized by tumor-specific traits of physical and functional integration absent in normal cells.^5^, ^34^, ^35^, ^36^ Furthermore, the assembly of these complex structures is modulated by the presence of PTMs, some of which only emerge in pathologic conditions that specifically underlie cancer or neurodegenerative diseases.^30^, ^37^

Importantly, over the last few years, cryo-EM has started shedding light on the ways Hsp90, and cochaperones and clients are organized in functional structures.^12^, ^13^, ^14^, ^15^, ^16^ Solution-state nuclear magnetic resonance approaches, on the other hand, revealed details of complex mechanisms of substrate sorting by cochaperones.^38^, ^39^, ^40^, ^41^, ^42^, ^43^

Despite this sophistication and impressive advancements, there is still no experimental technique that can provide atomic-level insights into the mechanistic details of chaperone regulation, interactions with partners, response to ligands, PTMs, and how this knowledge can inform new drug discovery efforts. To understand the correlations between chaperone structures, dynamics, and functions at an atomic level, we have little choice but to turn to theoretical approaches (Figure 2).

Indeed, theoretical methods to investigate chaperones fall into three main categories: (a) techniques aimed at investigating functional regulation mechanisms, from enzymatic activities to allosteric control; (b) approaches oriented to drug design; and (c) the emerging field of assembly formation, role of PTMs, and impacts on clients.

Here, we describe some of the main findings in the field of computational investigations of the Hsp90 code. We start from the study of the internal dynamics of the isolated protein in its different isoforms, report on how this knowledge can guide the discovery of small-molecule modulators and inhibitors, and close by discussing recent developments regarding the study of the supramolecular organization and dynamics of functional multiprotein complexes. In this framework, we will also discuss how the impact of PTMs on the various aspects of Hsp90 properties can be tackled *via* the use of computer simulations.

Finally, we will provide our perspective on how the appreciation of the rich and varied structural and dynamical personalities of Hsp90 and its network-forming attitudes can be exploited to discover new therapeutics and chemical tools for the investigation of the complex biology of chaperones.

## The internal dynamics of Hsp90 family members

Understanding how local protein perturbations, such as binding small-molecule ligands or introducing covalent modifications, can regulate functionally oriented (large-scale) motions of large multidomain proteins is still an open question in the field.^44^, ^45^, ^46^

**Fig. 3:**
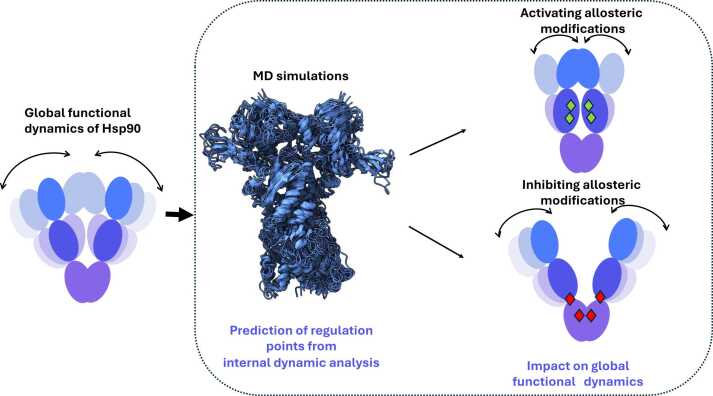
The internal dynamics of Hsp90 chaperone proteins. Simplified representation of the functional dynamics of Hsp90, its study by MD simulations, and the use of it for the design of modulators of activities. Modulators can be represented by mutations or small-molecule ligands targeting allosteric sites. Abbreviations used: Hsp90, heat shock protein 90; MD, molecular dynamics.

Simulative approaches have provided relevant insight into these themes in the case of Hsp90 (Fig. 2, Fig. 3): By analyzing and comparing atomistic simulations of different family representatives for which crystal structures of the full-length protein are available, namely mammalian Grp94, yeast Hsp90, and *Eschirichia coli* HtpG, several aspects of the internal dynamic regulation of the chaperone were revealed^44^ and, in follow-up work, experimentally validated.^46^ The chaperones were studied in complex with different nucleotide forms, ATP or Adenosine Diphosphate (ADP), as well as in the Apo state. Atomistic molecular dynamics (MD) simulations were run and analyzed with an original approach aimed to reveal coordinated internal fluctuation patterns and long-range communications. The data revealed that the three molecules share similar ligand-dependent structural modulations, which mostly consist of relative rigid-like movements of a limited number of quasi-rigid domains. Importantly, the domains and boundaries that were defined based on the analysis of fluctuations from atomistic MD revealed a subdivision of functional units different from the one obtainable from the crystal structure-based definition.

The analysis of pair-residue fluctuations also revealed two common primary hinges for the movements of these domains. The first hinge, whose functional role has been demonstrated by several experimental approaches, is located at the boundary between the NTD and middle domains. The second hinge is located at the end of a three-helix bundle in the middle domain and unfolds/unpacks going from the ATP state to the ADP state.

**Fig. 4:**
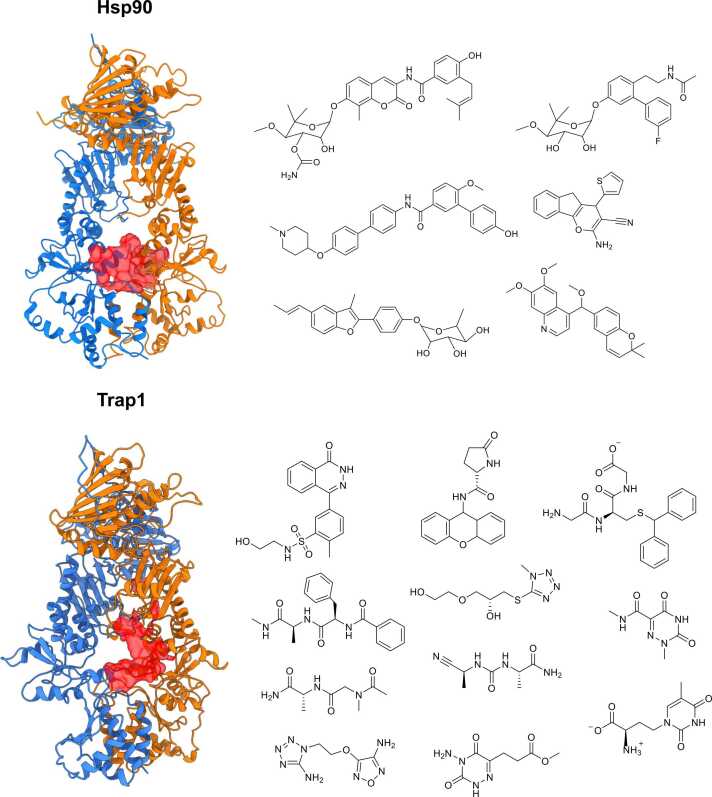
Selective targeting of Hsp90 isoforms *via* small molecules. The different structures and locations of allosteric sites in cytosolic Hsp90 (top) and mitochondrial Trap1 (bottom) are depicted together with the chemical structures of small molecules targeting them. Abbreviation used: Hsp90, heat shock protein 90.

This second site was proposed as a possible druggable site or regulation point to target for allosteric perturbation of the protein and tested both *via* small-molecule targeting and site-directed mutagenesis in the following papers (*vide infra*)^44^, ^46^, ^47^ (Fig. 3, Fig. 4).

Hsp90 structures and MD simulations were also used in combination with network analysis and network centrality methods to shed light on the statistical ensemble of allosteric interaction networks and communication pathways. Interestingly, the principal structurally stable communities emerging from network analysis are preserved in large dynamic changes characterizing different conformational ensembles.^48^ Structural stability analysis permitted to identify a network of conserved structurally rigid residues that could play a key role as mediators of allosteric communication and conformational changes. The framework of using atomistic simulations and network analysis was subsequently used also for elucidating the mechanisms of allosteric ligands, revealing a preference of different ligands for distinct states of the protein.^49^

MD-based analysis of nucleotide-based regulation of internal functional dynamics was next extended to the case of the mitochondrial isoform of Hsp90, namely TRAP1. Curiously, Trap1 was shown to crystallize in an original asymmetric conformation where one of the two protomers in the active state is buckled.^21^, ^50^ The asymmetry is due to the buckling of the region in the M-domain responsible for client binding.^21^, ^50^ The nucleotide-dependent internal dynamics of Trap1 were investigated by considering a number of nucleotide states, ranging from the full ATP or ADP states (the same nucleotide in both protomers) to all mixed states (nonhydrolyzed nucleotide in one protomer and the hydrolyzed one in the other), to the fully apo state.^51^ The results show a dynamic connection between the nucleotide state in the NTD and the asymmetric modulation of the dynamic and structural properties of the client-binding region in the M-domain. This region is the one that appears to be most responsive in terms of its conformational dynamics to nucleotide exchange. The results permit to develop a model whereby asymmetry actually determines differential and sequential hydrolysis steps for each protomer, with the buckled conformation favoring ATP processing (evidenced by an increased degree of internal coordination compared to other conditions), consistent with experimental structural, biophysical, and biochemical evidence. In this model, not only asymmetry does play a key role in regulating ATP hydrolysis, but it also expands the number of chaperone conformations that are presented to interaction with clients and cochaperones. Indeed, structural and dynamic asymmetry can be considered as a general feature of the Hsp90 family, which, on the one hand, helps regulate enzymatic activities and, on the other hand, favors the recruitment of a large number of diverse clients.^51^

Subsequently, a direct link was established between asymmetry and Trap1’s characteristic reactivity. A detailed computational investigation of Trap1’s potential ATPase mechanisms was run combining classical MD simulations and mixed quantum mechanical–molecular mechanical (QM/MM) methods.^52^

Classical MD simulations were used to monitor how frequently ATP and nucleophilic water can attain reactive poses compatible with nucleotide hydrolysis within the Buckled (Buc) and the Strained (Str) protomers. Semiempirical hybrid QM/MM coupled to umbrella sampling and benchmarked with density functional theory calculations were then used to sample reaction-free energy barriers within each protomer for two possible hydrolytic pathways. Enzyme-assisted hydrolysis, featuring a metaphosphate-like transition state and a catalytic glutamate deprotonating the nucleophilic water, is found to be favored in both protomers over substrate-assisted hydrolysis. However, it was also found that nucleophilic water sequestration by another water molecule bound to a vicinal tyrosine is rarer in Buc, proving that such a rare sequestration lowers reaction barriers for the enzyme-assisted pathway. The biologically significant tyrosine, which is a target of phosphorylating enzymes for regulation, is thus identified as the main mediator favoring ATP hydrolysis in Buc over Str, consistent with experiments.

These results provide molecular insights into the roles of structural asymmetry in the regulation of Trap1 and suggest that the asymmetry-generating Buckled region may be an allosteric target to modulate the functionally oriented aspects of Trap1 dynamics, opening new opportunities for the design of selective Trap1 chemical tools.^53^

Mader *et al.*^54^ used large-scale MD simulations and QM/MM calculations to investigate the couplings between the ATPase reaction in the active site and the global conformational dynamics of Hsp90. Importantly, they complemented computational investigations with site-directed mutagenesis experiments, Förster-resonance energy transfer, nuclear magnetic resonance spectroscopy, and small-angle X-ray scattering measurements. This endeavor generated a complete and overall consistent picture whereby the conformational switching in the protein, facilitated by a network of conserved ion pairs between the NTD, the domain that hosts the active site, and the middle domain, is seen to have a strong effect on the catalytic barrier of ATP-hydrolysis, mainly determined by electrostatic forces. The emerging model corroborates findings in which the long-range coupling between Hsp90 catalytic activities and the onset of motions related to possibly large conformational changes is at the basis of the chaperone’s functions and their regulation mechanisms. Interestingly, in simulations of the full-length Hsp90, the ion-pair opening is observed exclusively in only one of the two protomers, once again underlining the importance of structural and dynamic asymmetry in the regulation of this class of proteins.

The theme of asymmetry emerged also from the work of Alao *et al.*^55^ and Amankwah *et al.*^56^ who focused on Grp94 with a multiscale approach. First, the authors ran all-atom simulations of four different nucleotide-bound states, taking into account also mixed nucleotide conditions. Starting from the ATPase-competent structure, the authors found that the internal rigidity of the protein was maximal as Grp94 was bound to ATP. Consistent with previous models, the authors also revealed that ATP hydrolysis, as well as nucleotide removal, increased the dynamics of the ATP lid, ultimately abrogating interdomain communication. In an asymmetric condition induced by considering the hydrolysis of only one nucleotide, a more compact state was observed, consistent with experimental observations. The authors also identified potential electrostatic interactions between the long flexible linker and a helix in the M-domain where a cochaperone (namely BiP, the ER Hsp70) is known to bind. Interestingly, this interaction bridges the Hsp90 and Hsp70 chaperone cycles.^55^, ^56^

Finally, the authors employed normal-mode analysis and the structural perturbation method^57^ to identify residue communication pathways that may be important in the onset of the conformational change. Many of the identified residues turned out to be relevant for Adenosine Triphosphate binding, catalysis, and client binding. Consistent with previous findings, the obtained data revealed that Grp94, like other members of the Hsp90 family, is extremely responsive to the nucleotide state, *via* mechanisms based on allosteric rewiring, which ultimately facilitate conformational changes. Subsequently, interactions with BiP were probed experimentally *in vitro* and *in vivo*, showing that Grp94 interaction with the BiP chaperone system is required to fold only some clients and not others, suggesting that this chaperone cooperation may not be a general mechanism for Grp94.^56^

Important insight into Hsp90 structural evolution is currently being achieved *via* the integration of experimental data into MD simulations. In an elegant example, Hellenkamp *et al*.^58^ combined self-consistent networks of distance distributions obtained by Fluorescence Resonance Energy Transfer analysis with known domain structures. In this context, local structural dynamic changes are correlated with global conformational changes. This integrative approach allows the flexibility of multidomain Hsp90 to be explicitly shown and correlated with small-scale dynamics. Importantly, the authors access different conformational ensembles, namely the one corresponding to the closed X-ray structure, which comes from the averaging of the conformations visited in solution, and the one corresponding to the open state, which is characterized by interdomain fluctuations of up to 25 Å. Interestingly, the method permits to identify functional elements (hinges and domain boundaries/interfaces). In this work, MD simulations are an integral part of the method, providing an essential contribution to understanding the correlation of local and global dynamics of multidomain proteins, at a resolution which is not accessible with other techniques. In this framework, Sohmen *et al*.^59^ investigated the motions of full-length Hsp90 on a range of time scales, exploring the link between fast (local) fluctuations and slow global (allosteric) changes, with the former driving the onset of the latter. Fast dynamics on the hundreds of nanoseconds timescale are elucidated by a combination of single-molecule fluorescence, quasi-elastic neutron scattering, and all-atom MD simulations. The specific timescale of functional dynamic modes, such as interdomain motions, is shown to depend on the conformational state the Hsp90 dimer is populating. Furthermore, the authors show that the identified fast-scale motions are significantly influenced by the binding of cochaperones. The results of this integrative approach are consistent with previous endeavors that explored the role of the combination of short-time scale ordered motions in determining the onset and the selection of large conformational changes and helped provide a structure-dynamics-based model for the study of allostery.

Overall, the examples reported indicate that it is now possible to investigate the intricate relationships among nucleotide binding and processing, the selection of functionally oriented dynamic states for different regions of Hsp90, and the exploration of diverse conformational ensembles required to interact with clients and cochaperones. Most importantly, simulation-based data provide a basis to rationally guide new experimental endeavors, suggesting new strategies to design small-molecule regulators of Hsp90 as well as guiding the selection of targets for site-directed mutagenesis.

## Chemical control of the chaperone code

The knowledge of the molecular regulatory mechanisms of Hsp90 can be efficaciously exploited in the design of chemical modulators of the functions and interactions of this complex machine. Information obtained from dynamic investigations can, in fact, be translated into molecular design rules. In this framework, the privileged structures and dynamic states identified in different nucleotide conditions can be used as the basis to discover small molecules that selectively interfere with key functional substates of Hsp90. In principle, new molecular entities designed taking allosteric mechanisms into account can act as chemical switches that turn on or off specific cell functions by tweaking the internal dynamics of the target, thus allowing to tune and not only inhibit, the entire Hsp90-related signaling cascades that control cells.

These concepts were demonstrated in the discovery and rationalization of the activities of novel allosteric compounds targeting the region at the border between the M-domain and the CTD of Hsp90^47^ (Fig. 1, Fig. 4). This region was first identified by combining distance fluctuation analysis, which revealed residues distal from the ATP-binding site that are in allosteric communication with it, and pocket identification approaches. The latter was used to unveil, among sets of allosterically relevant residues, sites with the stereoelectronic features necessary to host a drug-like small molecule. On this basis, dynamic pharmacophore models were developed to screen drug libraries in the search for small molecules with the functional and conformational properties necessary to bind these “hot spot” allosteric sites. Importantly, the first experimental tests showed that the selected molecules bound the intended distal domain region and exhibited antiproliferative activity in different tumor cell lines, while not affecting the proliferation of normal human cells. Moreover, the identified compounds destabilized Hsp90 client proteins and disrupted association with several cochaperones known to bind the N-domain and M-domain of Hsp90.^47^

The initial hit was next characterized and further developed, starting from a structural and dynamic characterization of its complex with Hsp90.^60^, ^61^, ^62^ Simulations showed that the ligand could establish preferential interactions with a series of charged residues decorating the putative allosteric binding site. On this basis, complementary charged functionalities were installed on the first-generation ligands. Interestingly, all new leads showed stimulatory effects on Hsp90 ATPase and conformational dynamics. Activation mechanisms were probed by a combination of biochemical ATPase kinetic analyses, Fluorescence Resonance Energy Transfer analyses of Hsp90 closure kinetics, and MD simulations to understand the reorganization of dynamic states in the presence of allosteric ligands. Interestingly, the designed allosteric stimulators could perturb cochaperone and client-binding recruitment, ultimately impacting the viability of tumor cells. The designed chemical tools thus proved to act as allosteric activators of the chaperone that, at the same time, affected the viability of cancer cell lines for which proper functioning of Hsp90 is necessary.

The general model for the cellular impact of these compounds implies that the ATPase and conformational changes acceleration induced by the designed stimulators disrupt the kinetics of the chaperone cycle by modifying the delicate balance of timing and population distribution of Hsp90 structures that are presented to the other partners, which is necessary to assemble a correctly working folding machinery. The result is a perturbation of the efficiency of the complex and highly regulated Hsp90 chaperone system that requires the ordered and delicately balanced assembly of multiple factors.^61^, ^62^, ^63^

Consistent with this model, work by the Prodromou group^64^ showed that allosteric activation could be beneficial for the treatment of neurological disorders such as Alzheimer’s disease, in which the Aβ-peptide triggers tau hyperphosphorylation, formation of fibrils, and neurotoxicity. In this case, dihydropyridines were shown to upregulate the heat shock response specifically in diseased cells and provide a neuroprotective effect in an Alzheimer’s disease mouse model. The authors showed that, consistent with the aforementioned prediction models, the compounds predominantly targeted the M/CTD region of Hsp90, acting as stimulators of the chaperone’s ATPase activity. Interestingly, here, the site for dihydropyridines binding is confirmed by mutagenesis.

In a broader perspective, these papers showed the possibility to rationally design chemical tools that induce measurable perturbations in complex cellular systems, in terms of impact on the timing of conformational dynamics and the assembly of functional units. Such modulators, unlike classical orthosteric competitive inhibitors, which substantially aim to abrogate the whole functional spectrum of their target, can now be used to disentangle the intricacies of Hsp90 functions in their natural environment, complementing molecular and cell biology experiments where protein knock-outs, knock-down, or dominant negative mutants are typically employed. The use of specifically designed chemical tools could, in fact, permit to directly reconnect the observed perturbation on the system with the effect of the ligands on their target.

One additional advantage of allosteric design for the Hsp90 family is the possibility of selectively targeting isoforms. Indeed, allosteric sites tend to be under lower sequence-conservation and structure-conservation pressure than active sites, which are typically highly conserved, facilitating the design of specific ligands while reducing the risks of off-target engagement and consequently of toxicity or side effects.^65^

These concepts were demonstrated in the discovery of the first set of allosteric ligands, able to selectively target TRAP1, the mitochondrial paralog of the family.^53^ Biologically, it plays a key role in the regulation of energy metabolism, and its deranged activity has important implications in cancer, neurodegeneration, and ischemia. Starting from the characterization of the peculiar asymmetric dynamics of TRAP1 and its asymmetric patterns of long-range coordination, an allosteric pocket in the M-domain, distal to the ATP site, was identified as a suitable target for drug-like molecules. Small molecules were thus selected from a library with optimal stereochemical features to proficiently bind the putative allosteric pocket. The leads were demonstrated to inhibit TRAP1, but not cytosolic Hsp90, ATPase activity, and revert TRAP1-dependent downregulation of succinate dehydrogenase activity in cancer cells, blocking tumorigenic growth of neoplastic cells^53^ (Figure 4). Next, the characterization of the interactions and dynamic crosstalk between one of the active inhibitors and the protein were used as a basis for the development of a second generation of more potent ligands.^66^

The results described indicate that knowledge of the mechanisms of internal dynamics regulation can expand the chemical and functional space of chaperone ligands with wide therapeutic opportunities.

The models derived from the identification of putative allosteric sites were further exploited in the rationalization of the activities and in the optimization of a series of allosteric compounds, initially inspired by the natural product Novobiocin and introduced by the Blagg lab.^67^, ^68^, ^69^

Furthermore, the Blagg lab used simulations in combination with experimental photolabeling to explore the localization of allosteric sites.

One challenge still associated with allosteric inhibitors is the difficulty in obtaining co-crystallized complexes. MD simulations of the complexes of Hsp90 with allosteric ligands were shown on the one hand to provide important detail on their mechanisms of modulation of protein activities,^62^ while on the other hand to be actionable in the generation of pharmacophore models for virtual screening.^70^ In this context, Tomašič *et al.*^70^ used MD and ligand-based pharmacophores to generate hits with actual antiproliferative activities in MCF-7 and Hep G2 cancer cell lines.

Structure-based virtual screenings against the Hsp90β CTD binding site were further used to discriminate potentially viable ligands from a library of diverse compounds.^71^ Three selected virtual hits showed experimental activities with an IC_50_ in the low micromolar range against MCF-7 breast cancer cells. Next, these data were used to start an optimization process, delivering new analogs with significantly enhanced antiproliferative activities in MCF-7 breast cancer and SK-N-MC Ewing sarcoma cell lines.

One important open question in the search for allosteric modulators of Hsp90 (but this could hold for any ligand-modulated biological system) entails our ability to predict whether a selected compound will act as an inhibitor or an activator of the enzymatic activities of the chaperone.

Indeed, this aspect is particularly challenging for allosteric ligands. Indeed, experimental assays measure orthosteric function rather than ligand binding at the allosteric site, making the development of Structure Activity Relationships for allosteric ligands elusive if based on classical computational methods.^65^ In this framework, the advent of machine learning methods may be of great help in predicting and/or classifying the activities of a designed series of compounds. We provided examples of these concepts, integrating chemical information on the ligands, key descriptors of the dynamics of the chaperone, and the interactions in the complex to predict the allosteric effect of a wide and diverse class of ligands on the activation of Hsp90^72^ and TRAP1.^73^ While these endeavors are mainly initial ones, the results are encouraging and show that there is room for introducing these protocols in the selection processes for allosteric candidate drugs.

Summarizing, computational, and simulative approaches can open new opportunities to bridge our understanding of the fine mechanisms of regulation in Hsp90 with the design and selection of small molecules able to tune (and not simply inhibit) chaperone functions. Importantly, these approaches generate ligands that are able to discriminate between distinct isoforms, paving the way for the development of therapeutics that are selective for cancers where a certain isoform is overexpressed or overactivated while leaving the protein in healthy compartments unperturbed.

## Expanding the code: From single molecules to assemblies and the impacts of post-translational modifications

In general, the functions of the various members of the Hsp90 family in cells are not engendered by the single isolated chaperone. In the natural cell environment, functions emerge only when the chaperone combines with a series of task-specific regulatory proteins, the cochaperones, and the clients. The advent of cryo-EM has illuminated various multiprotein complexes comprising, for instance, Hsp90 and cochaperones or substrates.^10^, ^13^, ^14^, ^15^, ^16^, ^74^, ^75^, ^76^ The formation of complex multiprotein assemblies determines ATPase activities, substrate selection, substrate remodeling, and the timing and location where the chaperone cycle is to be realized. While this view of multiprotein complexes as the determinants of function is rapidly emerging,^77^ little is known about the details of their dynamic regulation, the roles of nucleotide processing, and the effects of the assembly of different complexes on the client in the different phases of the chaperone cycle.

**Fig. 5:**
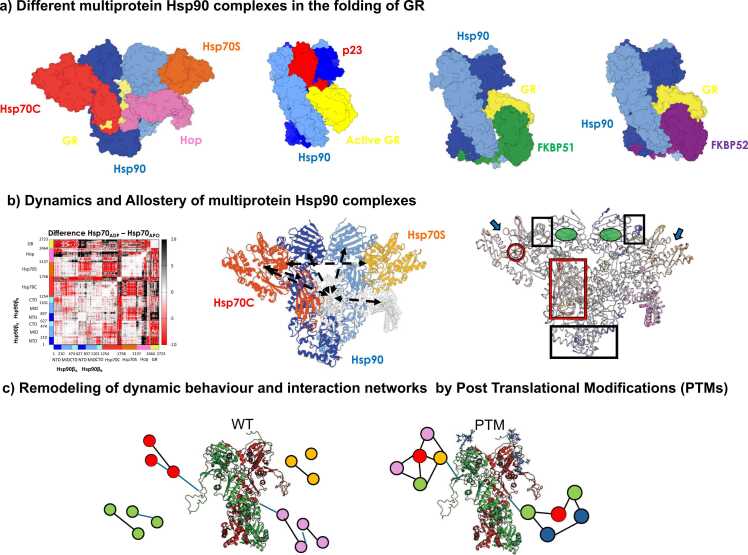
A summary of Hsp90 interactions and complex dynamics and implications for function. (a) Here, the different complexes are reported in which Hsp90 is engaged in the maturation of a substrate protein, namely GR, with different task-specific cochaperones. Structures are reproduced from PDB codes 7KW7, 7KRJ, 8FFW, 8FFV (b). The scheme for the analysis of the internal dynamics of complexes in the presence of different perturbations entails the characterization of internal coordination patterns at equilibrium and out-of-equilibrium responses to effector/client removal or modification (c). Modification of the internal dynamics of Hsp90 by post-translational modifications implies a remodeling of the interaction networks formed around the central chaperone. Abbreviations used: GR, glucocorticoid receptor; Hsp90, heat shock protein 90: PDB, Protein Data Bank.

The structures of assemblies revealed by cryo-EM have ushered in the possibility of studying the relationships between their structures, internal dynamics, and functional properties at high levels of resolution (Figure 5). The formation of multiprotein assemblies also determines the remodeling of chaperone structures and activities. Notably, the emergence of epichaperomes represents a gain-of-function for chaperones, where these stable and functionally distinct assemblies play critical roles in cellular homeostasis and disease states by integrating and coordinating the activities of multiple chaperones and cochaperones.^33^, ^36^, ^78^

Given the complexities and dimensions of the systems, multiscale approaches and network models have proved useful in shedding light on functional mechanisms. In this context, atomistic simulations combined with perturbation-based approaches, dynamic network modeling, and *in silico* deep mutational scanning helped identify the hot spots of protein stability and binding affinity in the Hsp90 complexes of Hsp90 with FKBP1 or P23 involved in glucocorticoid folding. Interestingly, such hot spots often coincide with the residues that relay allosteric messages in the Hsp90 dimer.^79^ These concepts were found valid also for the Hsp90–Hsp70–Hop–GR complex,^80^ where allosteric interactions between Hsp90, the client-bound Hsp70, and Hop cochaperone define allosteric residue clusters that control client recruitment (Figure 5).

Integrative approaches were also used to study the dynamic behavior of Hsp90 in complex with clients and the effects of client-induced allostery in the case of a kinase client^81^

Fully atomistic simulations of the Hsp90–Cdc37–Cdk4 complex dedicated to the maturation of kinase Cdk4 were combined with comparative analyses of long-range coordination and allosteric mechanisms as a function of the nucleotide states in the active site of the chaperone.^60^ The results reveal that nucleotide-dependent structural modulations reverberate in a striking asymmetry of the dynamics of Hsp90, which entails specific rearrangement in the regions proximal to the client-recruitment area. Moreover, the results identify specific patterns of long-range coordination between the nucleotide-binding site, the client binding pocket, the cochaperone, and the client. The model emerging from this work establishes a direct atomistic crosstalk between the ATP-binding site, the client region that is to be remodeled, and the surfaces of the Cdc37-cochaperone.

An open question on the functioning of folding complexes regards the possible ways in which different chaperones communicate and organize the assemblies for the remodeling and the efficient hand-over of the substrate at the various steps of the cycle. Using a combination of equilibrium and out-of-equilibrium MD simulation approaches, the mechanisms that underpin function in the glucocorticoid receptor (GR):Hsp90:Hsp70:cochaperone Hop client-loading and the GR:Hsp90: Cochaperone p23 client-maturation complexes were investigated.^82^ These assemblies are key in the folding cycle of the GR, a client strictly dependent upon Hsp90/Hsp70 for activity. The focus of the work was on getting insights into the processes by which the nucleotide-encoded message is relayed to the client and how the distinct partners of the assemblies cooperate to (pre)organize partially folded GR during loading and maturation. Different nucleotide states determine distinct dynamic profiles for the functional interfaces, defining the interactions in the complexes and modulating their overall flexibility to facilitate the progress along the chaperone cycle. The authors also show that the GR regions engaged by the chaperone machinery display specific energetic signatures in the folded state, which enhance the probability of partial unfolding fluctuations and facilitate the recruitment of the partially unfolded client into the chaperone machinery.

The results highlight the exquisite dynamic nature of chaperone assemblies and the conformational heterogeneity of their interactions: Dynamics, weak and heterogeneous interactions, and the assembly of different proteins in task-specific complexes, in fact, provide the basis for regulating the functions during the chaperoning cycle. Finally, it is worth noting that the concept of local unfolding propensities of clients has been actioned as the basis for the design of chemical disruptors of complexes, which act to block the folding and activation of specific proteins.^83^

Overall, the emerging picture is that of large systems that rely on a very delicate balance between structure, dynamics, and multiple interactions to carry out their functions in cells. Perturbing this balance can have effects that go well beyond the modulation of the conformational equilibrium or the reactivity of a single molecule. In this framework, PTMs have emerged as key perturbation factors capable of remodeling protein-interaction networks by modifying affinities and prompting Hsp90 to interact with different partners compared to its unmodified form. Through their impact on the mechanisms of assembly and the functions of large complexes, PTMs thus extend their influence to different scales up to the phenotypic level.

With the impressive advances observed over the last 10 years in proteomic technologies over the past 10 years, a huge number of PTMs have been uncovered on chaperones including phosphorylation, acetylation, methylation, SUMOylation, and ubiquitination. These modifications appear at different stages of cell life, involve different processes, and may be localized in specific compartments. As a consequence, collectively, they participate in determining the chaperone code.

While the identification of PTM sites is rather straightforward, the roles of the modifications are still not fully understood. Computational approaches represent ideal tools to start cracking the code and establish quantitative relationships between the presence of select PTMs and their role in chaperone functions. Using integrative approaches, it was shown that residues relevant as allosteric switch points could also be sites for PTMs.^44^, ^84^, ^85^

A notable example in the case of the mitochondrial isoform TRAP1 is represented by work from Papaleo *et al*.^86^ Using enhanced sampling simulations and *in-vitro* biochemical assays, the authors investigated the mechanisms induced by TRAP1 S-nitrosylation, showing how this covalent modification causes conformational changes that impact the activity spectrum of the chaperone. Interestingly, they extended their analysis to develop a workflow to identify targets that could sense the redox state of the SNO site. Finally, mutations in tumor suppressors or oncogenes were investigated, unveiling a connection between S-nitrosylation and a shift in the population of conformational ensembles that could ultimately be reconnected to functional variation.

Recent studies have started highlighting the importance of chaperone glycosylation. In particular, in Grp94, the Chiosis group identified a specific N-glycosylation pattern that alters Grp94 conformational fitness and favors a state most permissive for stable interactions with proteins at the plasma membrane.^37^ The glycosylation is thus responsible for changing the interaction spectrum of the chaperone, switching its functions from foldase to holdase. Glycosylation is furthermore significantly overrepresented in tumor cells.^35^, ^37^ Comparative simulative approaches were used here to compare Grp94 in the presence of different degrees of glycosylation and bound to different ligands, namely ATP, ADP, two different inhibitors, and the apo state.^34^ The results show that N-glycosylation at the specific positions observed in disease conditions (but not at others that appear devoid of pathologic consequences) modulates Grp94 internal dynamics and alters the conformational fitness of regions fundamental for the interaction with ATP and synthetic ligands. At the same time, pathologic glycosylations extend their effects long-range to modify the conformational dynamics of the client binding pocket. Conformational results were tested and validated experimentally with the use of Grp94 mutants, conformation-selective antibodies, and chemical probes.^34^, ^37^ This knowledge can be leveraged to design molecules that act on distinct glycosylation variants, targeting GRP94 disease-associated conformational states and assemblies, while leaving normal forms of the chaperone unscathed, such as demonstrated for the small molecule PU-WS13.^34^, ^37^, ^87^

Finally, it is worth noting that PTMs have been observed on cochaperones, further expanding the chaperone code.

As an example, the cochaperone Hop organizes and connects Hsp90 and Hsp70 in their multiprotein complexes. Alterations to Hop can thus lead to significant biological outcomes for chaperone mechanisms. Castelli *et al*.^88^ integrated simulations and experiments to unveil how Hop conformational fitness and interactions are actually modified by the phosphorylation of Hop residue Y354, a key hot spot that controls the mechanisms and populations of Hop open and closed states. The latter defines the recognition and binding of Hsp70/Hsp90. Phosphorylation or mutation of Hop-Y354 is seen to favor structural ensembles alternative to those observed in the GR maturation complexes that are indeed not optimal for stable interactions with Hsp90 and Hsp70. Experimentally, the consequence is the cellular accumulation of the stringent Hsp90 clients GR and the viral tyrosine kinase v-Src, with detrimental effects on their activities. This is a prime example whereby the effects of one single alteration are amplified at the cellular level through the perturbation of protein-interaction networks.

## Conclusions

Chaperones associated with normal and aberrant folding processes in cells are a significant target for fundamental and applicative studies. From the fundamental point of view, their investigation can unveil the details of the complex molecular interplay at the basis of cellular protein folding, a process whose fine implications still defy full characterization. From the applicative point of view, chaperones and their associated mechanisms provide a number of possibilities for therapeutic interventions in diseases that range from cancer to neurodegeneration.

In the specific case of Hsp90, multiple conformations, activation states, and a complex interaction landscape suggest that the protein has evolved as an adaptable player of stress regulation. In this context, while experiments mostly allow the measurement of steady-state and ensemble effects while not providing atomic-level resolution, atomistic simulations and computational approaches, in general, can add a new level of spatio-temporal detail and shed light on the complexities of the mechanisms of this fascinating system. Computation has proven useful in designing mechanism-based chemical tools, understanding the effects of PTMs on Hsp90 functional dynamics, and ushering in the possibility of directly looking at the regulation of chaperone assemblies. The continuously increasing computing power, combined with the advent of artificial intelligence structural prediction methods,^89^, ^90^, ^91^ promises to expand the role of computational/theoretical approaches even more in the next few years. New endeavors will expectedly entail the structural investigation of dynamic networks of interactions and how they are regulated by PTMs or small molecules, the mechanisms underlying their plastic remodeling in varying conditions, and their consequences on the organization of large supramolecular complexes that ultimately determine cell fates. Indeed, the function of these systems can be dictated by the nature of the ligands and PTMs, the range of conformations they adopt, and the interacting partners. These factors ultimately determine the specificity of the client substrate recruited for folding, as well as the timing and location required for their maturation and release. Using computers to crack these intricated questions of the chaperone code, with the role of the context explicitly taken into account, will help expand our knowledge into fundamental problems of cell survival and regulation, contributing to understanding the complexity of chaperone biology at different scales.

## Author contributions

**Giorgio Bonollo:** Writing – original draft. **Cristiano Sciva:** Writing – original draft. **Federica Guarra:** Conceptualization, Writing – original draft. **Giorgio Colombo:** Conceptualization, Funding acquisition, Investigation, Methodology, Project administration, Resources, Supervision, Writing – original draft. **Elisabetta Moroni:** Conceptualization, Funding acquisition, Investigation, Methodology, Project administration, Writing – original draft. **Gabriela Chiosis:** Conceptualization, Funding acquisition, Project administration, Writing – original draft. **Chiranjeevi Pasala:** Writing – original draft.

## Declarations of interest

The authors declare that they have no known competing financial interests or personal relationships that could have appeared to influence the work reported in this paper.

## Data Availability

No data were used for the research described in the article.
